# Clinical spectrum and outcome of nine patients with a novel genetic variant of galactosialidosis in the Kingdom of Bahrain

**DOI:** 10.1002/jmd2.12330

**Published:** 2022-09-04

**Authors:** Zahra Alsahlawi, Emtithal Aljishi, Ammar Kheyami, Ahmed Alekri, Sayed Mohammed Jawad Alwedaie

**Affiliations:** ^1^ Metabolic Diseases and Clinical Genetic, Department of Paediatrics Salmaniya Medical Complex Manama Kingdom of Bahrain; ^2^ Department of Paediatrics Arabian Gulf University Manama Kingdom of Bahrain; ^3^ Royal College of Surgeons in Ireland–Medical University of Bahrain Busaiteen Kingdom of Bahrain

**Keywords:** galactosialidosis, lysosomal storage diseases, pediatric metabolic diseases

## Abstract

Galactosialidosis (GS, OMIM #256540) is a systemic autosomal recessive disorder that is due to a mutation in the cathepsin A (CTSA) gene. Its worldwide prevalence is rare, accounting for ~146 cases reported cases globally. In Bahrain alone, nine cases have been confirmed. This article aims to shed a light on the clinical spectrum and outcome of these nine patients who share the same novel genetic mutation. The article was written retrospectively based on the review of patients' medical records, which included clinical notes, biochemical, radiological, and genetic assessments. Analysis of the data from all nine patients revealed that the diagnosis was most commonly made at the early years of life. As expected from any systemic disorder, the disease affects multiple organ systems with musculoskeletal and the gastrointestinal system being most commonly involved. Short stature, skeletal deformities, coarse facial features, and different degrees of hepatomegaly are among initial presentations of the disease. Notably, one of the patients described in this article, developed severe form of cardiomyopathy and another one, presented with nonimmune hydrops fetalis, both of which considered rare occurrences in the context of GS. Genetically, all patients had the similar genetic mutation confirmed by laboratory tests. A few patients have had their diagnoses made based upon family history alone.

## 1 INTRODUCTION

Galactosialidosis (GS, OMIM #256540) is an autosomal recessive lysosomal storage disease that was first reported in 1971 by Goldberg et al.^1^ The underlying biochemical cause is a deficiency of β‐galactosidase and α‐neuraminidase.^2^, ^3^ These two enzymes form a complex with a lysosomal protective protein known as cathepsin A.^3^ The disease is due to a mutation in the cathepsin A (CTSA) gene, localized on 20q13.12.^3^ Most of these mutations are missense/nonsense mutations. Patients with GS display a wide range of clinical manifestations and are classified into three types according to the age of onset and severity of their symptoms. The three types include early infantile (EI), Late Infantile (LE), and Juvenile/Adult (J/A). GS affects multiple organ systems including the central nervous system, cardiovascular system, gastrointestinal system, eyes, and musculoskeletal system. Involvement of integumentary system leads to angiokeratomas, telangiectasias, and hemangiomas, while vacuolated lymphocytes, anemia, and thrombocytopenia are manifestations of hematopoietic system involvement. The EI type manifests as hydrops fetalis, while the LE type presents with hepatosplenomegaly, cardiac, and neurological signs. The juvenile forms often present with angiokeratoma, myoclonic jerks, and neurological deterioration. Unusual complications like inflammatory arthritis have also been observed in GS patients.^4^ Although a number of clinical trials have been performed in an attempt to find a curative method for the disorder,^5^ no treatment option has yet been introduced to the clinical practice and therapy is limited to supportive care.

Up until the present time, nearly 146 cases of GS were reported worldwide.^3^, ^4^, ^6^, ^7^, ^8^ In Bahrain alone, nine cases have been diagnosed, which places the country among those with high GS prevalence rate. In this study, we retrospectively reviewed the clinical presentations and major complications observed in nine Bahraini patients from four different families with GS, all of which share the same novel genetic variant.

## 2 MATERIAL AND METHOD

This was a retrospective chart review study, conducted in department of pediatrics in Salmaniya medical complex, the only tertiary care hospital that provides clinical metabolic care in Bahrain. Between January 1998 and February 2022, all patients with a confirmed diagnosis of GS, have been enrolled in the present study. Diagnostic methodologies included clinical, biochemical, radiological, and genetic assessments. The genetic tests were conducted in BioScientia International laboratory Center in Frankfurt, Germany. All diagnostic tests and findings were discussed in a multidisciplinary team prior to a decisive diagnosis.

Patients' medical records (including both paper and electronic system) were used to retrieve all the necessary patients' details encompassing the demographic and clinical information. This study was ethically approved by the Research Ethics Committee of Government Hospitals in Bahrain (IRB number: 32060322). The study was conducted in accordance to Helsinki declaration principles.

For better identification of patients with their clinical characteristics, each subject has been given a specific number.

## 3 RESULTS

From January 1998 to April 2022, 332 patients have been identified with inborn errors of metabolism. Among these, 56 were diagnosed with lysosomal storage disorders and only nine of them were confirmed to be affected by GS. A total number of nine patients, comprised of six females and three males, were found from four different families. There was a relatively large variation in the age of the patients at the time of diagnosis, with the youngest one being diagnosed at birth and the oldest one at 50.

**FIGURE 1:**
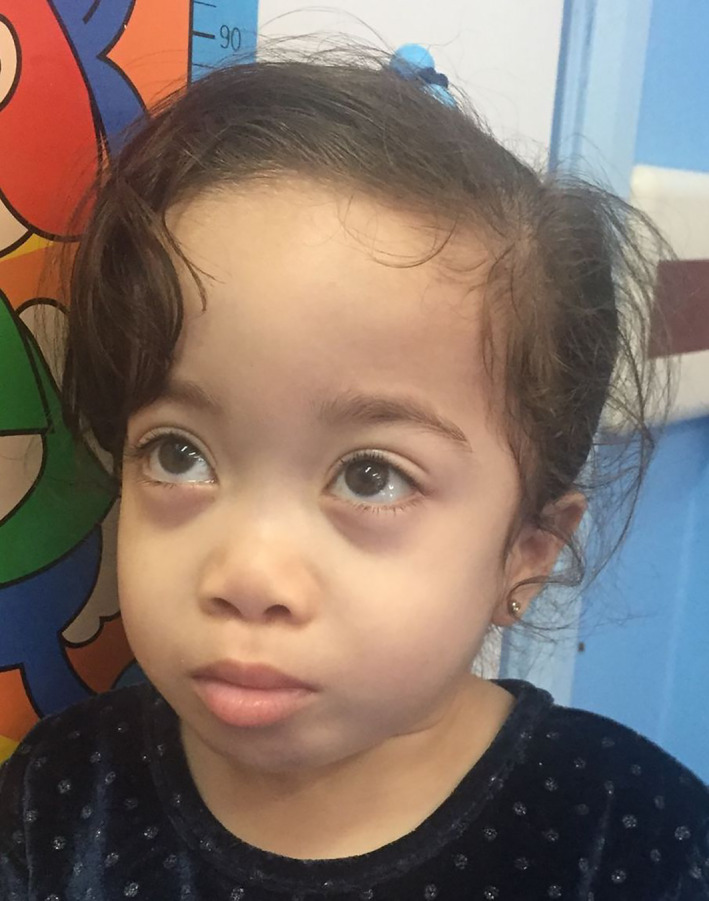
Typical facial features of GS (Patient‐7)

**FIGURE 2:**
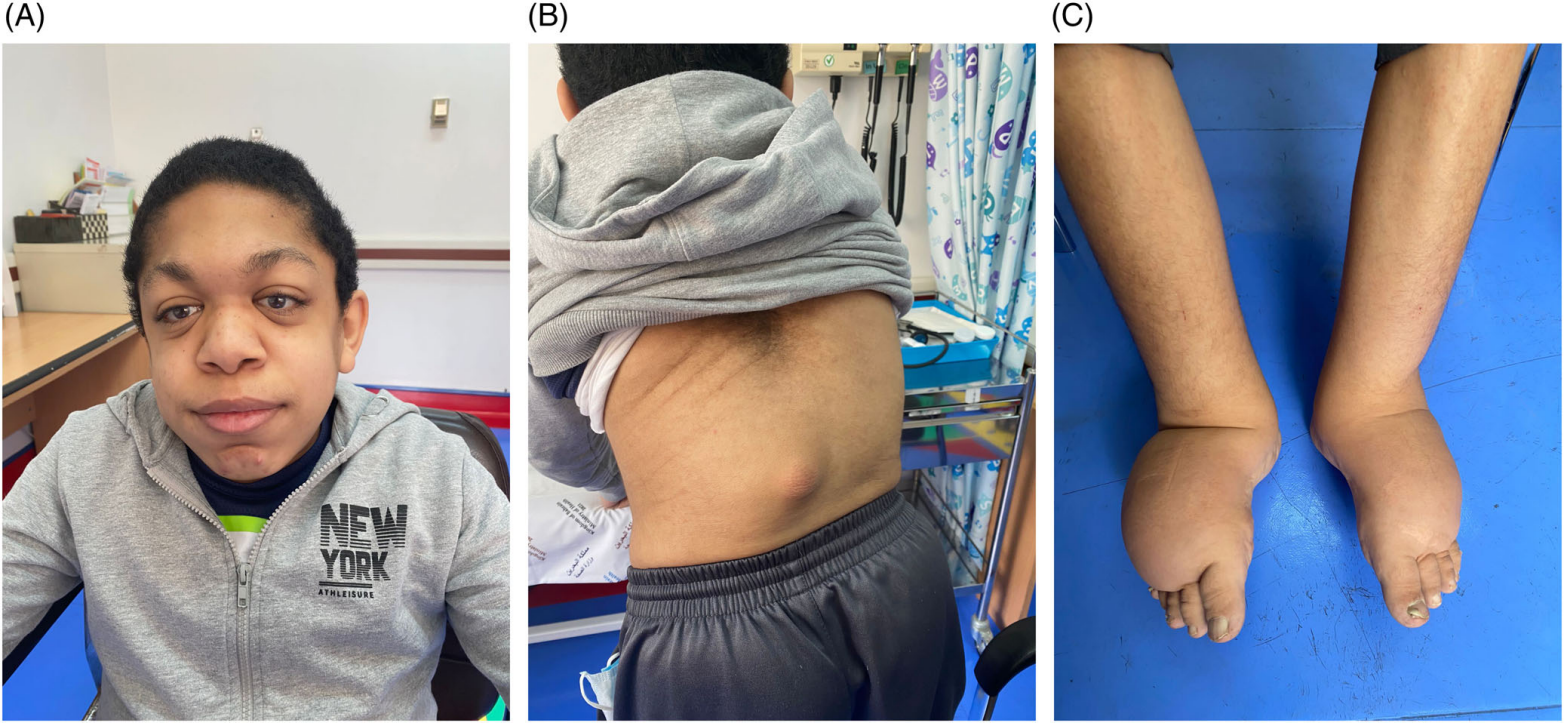
(A) Typical facial features of GS (Patient‐3). (B) Thoracolumbar kyphosis (Patient‐3). (C) Bilateral lower limb lymphedema (Patient‐3)

A summary of patients' demographic and clinical data can be found in Table 1. Most of the patients were diagnosed at the early years of life. Four patients were born by normal vaginal delivery and for the rest of the six patients, the mode of delivery was not mentioned in the records. Patient‐2, who was born prematurely at 26 weeks gestational age, was admitted to NICU for nonimmune hydrops fetalis, extremely low birth weight (1.04 kg), interventricular hemorrhage, respiratory distress syndrome, and necrotising enterocolitis. Short stature was the commonest presenting sign along with skeletal deformities and coarse facial features. Patient‐9, who was diagnosed with the condition at the age of 41, contracted a severe form of COVID‐19 infection in 2021, which was exacerbated by respiratory pneumonia that led to her death eventually. From Gastrointestinal point of view, all patients developed hepatomegaly at some points with Patient‐1 being affected by Gastro‐esophageal Reflux Disease, evident on barium swallow study. Patient‐9 suffered from supra‐umbilical and an obstructed left inguinal hernia, which was operated on 2019. In relation to cardiovascular disorders, valvular heart diseases were observed in Patients 1 and 8 with mitral valve being most frequently involved. Of note, Patient‐7 was diagnosed with atrial septal defect, ventricular septal defect and left ventricular hypertrophy. Her last echocardiography study (December 2019) revealed a dilated and poorly contractile left ventricle, with systolic function of 7% and ejection fraction of 25%. Later on, this patient developed severe dilated cardiomyopathy, which led to her death at the age of seven. Patient‐3 developed congestive heart failure with his last echocardiography study showing ejection fraction of 38%. Neurologically, hypotonia was observed in Patient‐7 (Figure 1), while an episode of febrile seizure was seen in Patient‐1 at the age of five. No episodes of dystonia or acute encephalopathy have been observed in any patient till the present time. Fundoscopic examinations were done for three patients only (Patients 3, 4, and 7), all of which were negative for cherry red spots. Of note, five patients developed puffy eyelids at the early years of life (Patients 1, 3, 4, 6, and 7) with Patient‐9 suffering from corneal clouding. Angiokeratomas (which are multiple blue‐red hyperkeratotic papular skin lesions) were seen along the limbs and back of all patients, in addition to depressed nasal bridges (seen in all patients as well). Features of dysostosis multiplex including thoracolumbar kyphosis, large skull with a thickened calvarium and bullet‐shaped phalanges were observed among all nine patients. Patient‐3 developed severe proteinuria and bilateral lymphedema of the lower limbs (Figure 2A–C). Subsequent renal pathological studies demonstrated focal and segmental glomerulosclerosis in addition to mild, focally moderate interstitial fibrosis, and tubular atrophy. This patient underwent an MRI of the brain and spine at the age of 10 and was diagnosed with L1 hemivertibra with focal kyphosis and central spinal canal stenosis. MRI of the brain in particular illustrated signal abnormalities in the area of globus pallidus bilaterally.

**TABLE 1:** Summary of the patients' demographic and clinical data

		Family 1		Family 2	Family 3				Family 4	
		Patient 1	Patient 2	Patient 3	Patient 4	Patient 5	Patient 6	Patient 7	Patient 8	Patient 9
Current Age (years)		20	5	18			44			53
Gender (Male, Female)		Female	Male	Male	Female	Male	Female	Female	Female	Female
Age at diagnosis (years)		5	At birth	6	8	16	30	6 months	41	50
Status (Alive, Expired)		Alive	Alive	Alive	Expired at 25	Expired at 16	Alive	Expired at 7	Expired at 44	Alive
Cause of death					Severe respiratory failure	Post spinal surgery		Severe dilated cardiomyopathy	Severe pneumonia post COVID‐19 infection	
Initial presenting symptoms	Short stature	++	++	++	++	++	++	++	++	++
	Coarse facial feature	++	++	++	++	++	++	++	++	++
	Heart murmur	++	++	++	++	++	++	++	++	++
	Hepatomegaly	++	++	++	++	++	++	++	++	++
	Prematurity		++							
	Nonimmune hydrops fetalis		++							
	Positive family history in the sibling		++							
	Skeletal deformities			++						
	Dysostosis multiplex	+	+	+	+	+	+	+	+	+
	Angiokeratoma	++	++	++	++	++	++	++	++	++
Previously affected relative			+			+	+	+		+
Genetic mutation		CTSA (homo) c.607C>A, p.Pro203Thr	CTSA (homo) c.607C>A, p.Pro203Thr	CTSA (homo) c.607C>A, p.Pro203Thr	Not done	Not done	CTSA (homo) c.607C>A, p.Pro203Thr	CTSA (homo) c.607C>A, p.Pro203Thr	CTSA (homo) c.607C>A(p.Pro203Thr)	Not done
		Positive Sanger sequencing	Positive Target mutation	Positive Sanger sequencing			Positive target Mutation	Positive Sanger sequencing	Positive Sanger sequencing	

**FIGURE 3:**
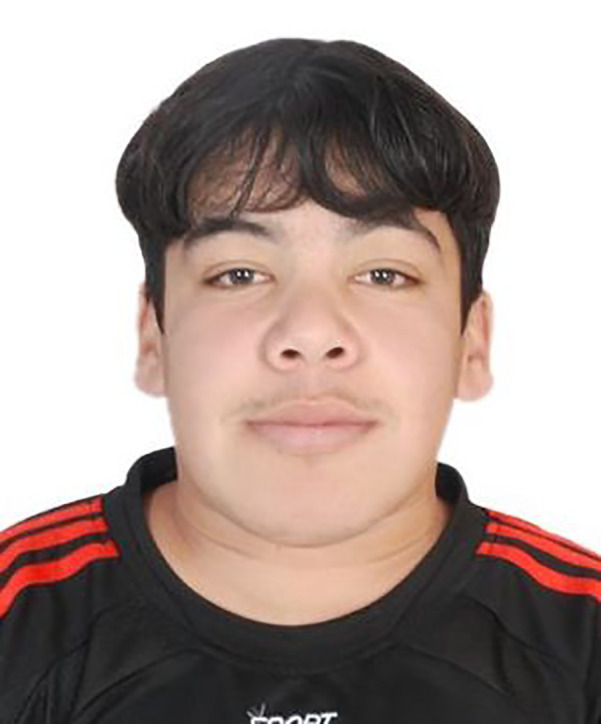
Typical facial features of GS (Patient‐5)

Four out of nine patients were expired, including Patient‐5 (Figure 3) who died of post spinal surgery sequela and Patient‐7 who lost her life due to severe dilated cardiomyopathy. The other five patients are still alive.

## 4 GENETICS

From a genetic stand point, the same CTSA mutation was found across all four families described in this study, however, not all patients we were able to undergo the genetic test. In Family 1, Patient‐1 had a sanger sequencing for the CTSA gene, resulted in finding a homozygous mutation c.607C>A(p.Pro203Thr). This genetic alteration leads to the substitution of threonine for the wild type proline at amino acid 203. Her brother (Patient‐2) who had an early presentation, underwent a targeted mutation analysis and showed the same finding with homozygous mutation c.607C>A(p.Pro203Thr) in CTSA gene. Patient‐3 in Family 2 also demonstrated a positive molecular testing by finding a homozygous nucleotide exchange c.607C>A in exon 6 of the CTSA gene. In Family 3, which is comprised of four affected members, Patients 4 and 5 had had their diagnosis based on specific clinical, skeletal and biochemical findings, although they both expired without a genetic testing. Patient‐6 (their sister) and Patient‐7 (their niece) had a molecular genetic testing of CTSA gene and the homozygous mutation c.607C>A(p.Pro203Thr) was found in both. The parents of Patient‐7 were tested for the mutation and they turned out to carrying the same mutation in a heterozygous state. Finally, in Family 4 the sanger sequencing was performed on Patient‐8 and the result confirmed GS by finding the mutation c.607C>A(p.Pro203Thr) as homozygous in CTSA gene. Her sister (Patient‐9) did not have a genetic test and her diagnosis was based on the same clinical findings that were found in her affected sister. According to American College of Medical Genetics and Genomics, the new variant described for the above patients was considered class 3, namely variant of uncertain significant. The electropherogram results of Sanger sequencings were difficult to retrieve, given that all the genetic tests were performed abroad and several years have passed since the results were received.

## 5 DISCUSSION

This retrospective chart review strived to present clinical features of nine patients diagnosed with GS retrospectively and to report a novel genetic mutation as a founder effect in the Bahraini population. to the best of the authors' knowledge, there has only been one reported case of GS in the whole middle east.^9^

Although the molecular analysis of the CTSA gene was not performed in all nine patients, a positive mutation in at least one patient from each family, could be sufficient for the diagnosis of the nontested patients. The same homozygous mutation c.607C>A(p.Pro203Thr) was found across all four families described. This mutation is possibly deleterious, since the proline residue at position 203 is highly conserved. Nine out of ten used bioinformatics programs have predicted this variant to be damaging. Mutation Taster (www.mutationtaster.org) calls the variant “disease causing,” since it affects the highly conserved amino acids. Although the authors were fully aware of the importance of functional studies (e.g., insilico protein modeling, mRNA expression, etc.) for proving the significance of such novel genetic mutations, given the lack of proper research facilities in our hospital, this could not be fulfilled.

Up to the date of writing the present article, 35 CTSA genetic mutations linked to GS have been published.^8^ To the best of our knowledge, the mutation described in this study has not been previously reported in the literature and could be regarded as a founder mutation in the Bahraini population.

As a rare genetic disorder, GS has remained a largely unknown disease entity, with most of our knowledge coming from individual reports (Table 2). Ethnically speaking, 40% of affected individuals are from Japanese origins, followed by 4% Portuguese.^3^ According to a recent review, the mean age at diagnosis has been 20.2 years.^3^ However, the same parameter is different among various disease subtypes. The overall survival rate for EI, LE, and juvenile types has been reported to be 48 years.

**TABLE 2:** Summary of findings of similar studies, including the findings of our nine patients

Study author, type of study	Number of patients (*n*)	Form of the disease	Age of presentation (years)	Age of diagnosis (years)	Unusual complications	Gene mutation
Alsahlawi et al. (2022)	9	EI/J/A	Range from birth to 50 years	Range from 1 month to 50 years	Severe renal fibrosis, lower limb lymphedema, severe dilated cardiomyopathy	CTSA (homo)
						c.607C>A(p.Pro203Thr)
Verkuil et al.^4^	1	J/A	17 months	17 months	Inflammatory arthritis	
Fukuyo et al.^6^	1	J/A	35	35	abnormalities of the outer retina	homozygous mutation in the CTSA gene–IVS7+3A>G
Libbrecht et al.^7^	1	EI		3 days		(c.265A>C, p.Ser89Arg)
Nakajima et al.^8^	1	J/A	23	23		Compound heterozygous mutation consisting of NM_00308.3(CTSA):c.746+3A>G and c.655‐1G>A
Kartal et al.^10^	1	EI	At birth	At birth		Homozygous mutation (c.1284delG)
Okulu et al.^11^	1	EI	At birth	At birth	Secondary hyperparathyroidism	p.F191Pfs*39 (c.569_570delTT)
Caciotti et al.^12^	4	EI		Range from 1 months to 14 months		c.1216C>T(p.Gln406), c.775 T>C (p.Cys259Arg), c.347A>G (p.His116Arg)

Patients with EI types usually present within the first 3 months of life with their cardinal symptoms being hepatosplenomegaly, ascites, edema, and fetal hydrops. Telangiectasias are specifically observed among the EI type of patients. Most of the cases in the present study are EI type.

LI patients are identified by the first year of life and the characteristic clinical features include hepatosplenomegaly, cardiac involvement, dysostosis multiplex, as well as hearing, and visual impairments. Presentation with knee Inflammatory arthritis has recently been reported in an LI GS patient,^4^ with myoclonus and ataxia being classified as rare presentations in the context of LI GS.

J/A type accounts for majority of GS patients and the symptoms usually appear during the adolescent years. The clinical picture includes myoclonus, ataxia, seizures, mental retardation, and visual impairments; however, in some cases, the manifestations could be of mild and limited origin.^6^ Angiokeratoma of the skin is exclusively found among J/A patients.

In our study, two patients (2 and 7, respectively) developed major complications including renal fibrosis, lymphedema and severe dilated cardiomyopathy, all of which have rarely been reported among GS patients in the literature.

Regarding the genotype phenotype correlation, it is worth mentioning that although the genotype of all the patients has been the same, the phenotypes have been quite different, given the various presenting sign and symptoms that each patient exhibited.

From a therapeutic point of view, treatments have mainly focused on supportive care and management of complications. One recent clinical trial has evaluated the long‐term efficacy and safety of adeno‐associated virus (AAV)‐mediated in vivo gene therapy in model mice affected by GS, which held promising results.^5^

## 6 CONCLUSION

Here, we reported a novel genetic mutation of GS for the first time in the Kingdom of Bahrain, a mutation that has never been reported before which can be accounted as a founder effect in the country. We also reported the occurrence of three severe complications that have rarely been associated with GS. Given all these findings, we prompt clinicians to be vigilant of these complication and plan the proper management in advance.

## CONFLICT OF INTEREST

Zahra Alsahlawi, Emtithal Aljishi, Ammar kheyami, Ahmed Alekri, and Sayed Mohammed Jawad Alwedaie declare that they have no conflict of interest.

## INFORMED CONSENT

Additional informed consent was obtained from all patients for whom identifying information is included in this article.

## Data Availability

The genetic data provided in this article are available in a printed paper form. They are all confidential files and can only be accessed by the consultant responsible for this article; however, we can provide you with the images of these paper documents upon your request.
